# Supplementary material for: Evaluation and implementation of triple‐channel radiochromic film dosimetry in brachytherapy
Source: J Appl Clin Med Phys. 2014 Jul 8;15(4):280–96. doi: 10.1120/jacmp.v15i4.4854 (PMC5875501; doi:10.1120/jacmp.v15i4.4854)
Supplement: Supplementary file 1 — Supplementary Material [file ACM2-15-280-s001.docx]

Evaluation and implementation of triple-channel radiochromic film dosimetry in brachytherapy

**Antony L Palmer,^1,2,a^ David Bradley,^1^ Andrew Nisbet,^1,3^**

*Department of Physics,***^1^** *Faculty of Engineering and Physical Science, University of Surrey, Guildford, Surrey, UK*

*Medical Physics Department,***^2^** *Portsmouth Hospitals NHS Trust, Portsmouth, Hampshire, UK*

*Medical Physics Department,***^3^** *Royal Surrey County Hospital NHS Foundation Trust, Guildford, Surrey, UK*

Corresponding author: Antony L Palmer,^a^

*Medical Physics Department,
F-Level,
Queen Alexandra Hospital,
Portsmouth Hospitals NHS Trust,
Portsmouth, PO6 3LY, UK
Tel.: +44 (0)2392 286000*Email: antony.palmer@porthosp.nhs.uk

Submitted: December 28, 2013

Accepted: February 24, 2014

Running title: Triple-channel film dosimetry in brachytherapy

Evaluation and Implementation of Triple-Channel Radiochromic Film Dosimetry in Brachytherapy

**Abstract:** The measurement of dose distributions in clinical brachytherapy, for the purpose of quality control, commissioning or dosimetric audit, is challenging and requires development. Radiochromic film dosimetry with a commercial flatbed scanner may be suitable but careful methodologies are required to control various sources of uncertainty. Triple-channel dosimetry has recently been utilised in external beam radiotherapy to improve the accuracy of film dosimetry, but its use in brachytherapy, with characteristic high maximum doses, steep dose gradients and small scales, has been less well researched. We investigate the use of advanced film dosimetry techniques for brachytherapy dosimetry evaluating uncertainties and assessing the mitigation afforded by triple-channel dosimetry. We present results on post-irradiation film darkening, lateral scanner effect, film surface perturbation, film active layer thickness, film curling, and examples of the measurement of clinical brachytherapy dose distributions. The lateral scanner effect in brachytherapy film dosimetry can be very significant, up to 23% dose increase at 14 Gy, at ±9 cm lateral from the scanner axis for simple single-channel dosimetry. Triple-channel dosimetry mitigates the effect, but still limits the useable width of a typical scanner to less than 8 cm at high dose levels to give dose uncertainty to within 1%. Triple-channel dosimetry separates dose and dose-independent signal components and effectively removes disturbances caused by film thickness variation and surface perturbations in the examples considered in this work. The use of reference dose films scanned simultaneously with brachytherapy test films is recommended to account for scanner variations from calibration conditions. Post-irradiation darkening, which is a continual logarithmic function with time, must be taken into account between the reference and test films. Finally, films must be flat when scanned to avoid Callier-type effect and to provide reliable dosimetric results. We have demonstrated that radiochromic film dosimetry with Gafchromic EBT3 film and a commercial flatbed scanner is a viable method for brachytherapy dose distribution measurement, and uncertainties may be reduced with triple-channel dosimetry and specific film scan and evaluation methodologies.

**Key Words:** Film dosimetry, Gafchromic EBT3, triple-channel dosimetry, high dose rate (HDR) brachytherapy, quality control

**PACS number:** 87.55.Qr, 87.56.bg, 87.55.km

**I. INTRODUCTION**

The measurement of dose distributions produced by clinical brachytherapy treatment equipment is challenging due to large dose ranges, high dose gradients and small spatial scales. However, accurate dose verification is required to confirm that the intended radiation treatment prescribed is actually delivered. Radiochromic film dosimetry is often employed for dose distribution measurement in radiotherapy, with a number of advantages over other dosimetry methods, including high spatial resolution, low energy dependence and near water equivalence,^(1, 2, 3, 4)^ and relative ease of signal readout with a desktop scanner.^(5, 6)^ However the resulting pixel value in a scanner-produced image of a radiochromic film is a complex convolution of scanning lamp emission, absorption of the film, sensitivity of CCD array, and importantly optical properties of the scanner along the light path, influenced by polarisation caused by the film. All of these may change as a function of position on the flatbed scanner, and each have further dependencies including scanner warm-up characteristics and fluctuations, film orientation, film temperature, film humidity level, post-irradiation film darkening kinetics, dose-dependent effect on polarisation, scan and analysis protocols. There are likely to be large dose variations across films used in the dosimetry of brachytherapy applications, which may exacerbate the above issues. For brachytherapy dosimetry Perez-Calatayud et al.^(7)^ reports that radiochromic film must be considered “under development at this time because of numerous artefacts which require rigorous correction”. Advanced film dosimetry techniques, including triple-channel scan processing,^(8, 9)^ has the potential to improve the accuracy and reliability of film dosimetry and mitigate sources of uncertainty. The use of all three colour channels of a flatbed scanner has been proposed to correct for deviations from the calibrated average film-scanner response. Hence the non-dose dependent signal component can be separated from the signal and compensated for. The triple-channel film dosimetry implemented by Ashland Inc, NJ, USA,^(8)^ is stated to have the following features and advantages (a) separates dose and dose-independent parts of the signal and signal disturbances such as film thickness variation, scanner distortion, and background correction, which can then be accounted for, (b) enables entire dynamic dose range of the film, (c) improvement of dose map accuracy, and (d) indicates any inconsistencies between film and calibration and estimates the dose uncertainty of the measurement. Triple-channel film techniques have been studied in external beam radiotherapy applications, typically up to 2 to 3 Gy,^(10, 11)^ but their application in brachytherapy, with prescription doses of 7 to 8 Gy and peak doses that are significantly higher, as well as different dose distribution and different energy spectrum, needs further investigation.

In brachytherapy, film dosimetry applications are usually for routine quality control, commissioning or audit,^(12)^ where there is generally some freedom in the time between irradiation and scanning. However, post-irradiation film darkening kinetics is often considered a potentially significant uncertainty in film dosimetry.^(13)^ Polymerization of the active component in radiochromic film continues after irradiation, but the rate of polymer growth decreases with time. Borca et al.^(14)^ studied post irradiation darkening appropriate for external beam radiotherapy, up to 4 Gy over 3 days. The effect is studied in the present work at dose levels and scan times appropriate for brachytherapy applications, up to 14 Gy, and we evaluate darkening kinetics during a 3 month post-irradiation period. Irrespective of whether multiple or single channel dosimetry is used, it is important to characterise post irradiation darkening to minimise uncertainties in film dosimetry.

Characteristics of film dosimetry that must be understood for accurate use have been covered in the literature, including film orientation, batch consistency and disabling scanner image correction.^(1, 15)^ These considerations are not repeated in the current work. There are however factors that have not been sufficiently researched nor their impact in brachytherapy applications assessed: We investigate post-irradiation darkening, lateral scanner effect, film surface perturbation, film active layer thickness, measurement of clinical brachytherapy dose distributions, film curling, and proposed film dosimetry methodologies, including where appropriate, whether triple-channel dosimetry can improve dosimetric accuracy. A potentially significant artefact is the response of the scanner to the film as a function of lateral distance on the scan plane, which increases with dose level, described by Menegotti et al,^(16)^ in the range 0 to 7 Gy. The response artefact is caused by the polarization of transmitted light by the near-linear array of polymer rods in the film, and the varying transmission on reflection at mirrors in the scanner being a function of angle for polarised light, which changes with lateral position on the scanner. The longer wavelength red light is affected significantly more than green and blue light, hence it is expected the multi-channel process will mitigate the artefact. In this work we investigate the effect at up to 14 Gy, appropriate for brachytherapy, and compare the calculated film-dose using single- and triple-channel dosimetry.

Inadvertent surface contamination such as finger print grease and scratches from contact with phantoms and jigs for example, can cause artefacts in film dosimetry, changing the optical density in the scanned image. The measured density will also vary proportionally with the thickness of the active layer, which for Gafchromic EBT3 is 28 microns. Manufacturing tolerances of the active layer thickness are typically up to 1.5% which would result in an uncertainty in reported dose of 1.5% for single channel conventional dosimetry. Triple-channel film dosimetry is expected to mitigate such dose-independent signal components. We compare the response from single- and triple-channel dosimetry in the presence of such film surface perturbations, and extreme active layer thickness variations, in doses typical for brachytherapy applications.

A methodology for efficient dosimetry using radiochromic film has been proposed by Lewis et al.^(17)^ in which test films are scanned together with a reference dose film strip and an unexposed film strip, in order to eliminate by normalizing any scan-dependent uncertainty such as scanner lamp output. This protocol is evaluated for external beam radiotherapy (IMRT and VMAT) by Lewis et al.^(17)^ We extend the evaluation to typical clinical brachytherapy situations and suggest a modification.

In this work we evaluate the perceived advantages of triple-channel compared to single-channel analysis in dosimetric test situations applicable for brachytherapy. We also use the latest Gafchromic EBT3 film, which is structurally different to its predecessors, EBT and EBT2; the latter having had greater coverage in the published literature to date.^(18)^ We also compare measured film dose maps for brachytherapy exposures with brachytherapy treatment planning system intended dose distributions, calculated using single- and triple-channel film dosimetry, to evaluate any benefit of increased film dose range and dose map accuracy of the triple-channel technique. Finally, taking account of the above work, we propose an optimum methodology for film dose distribution measurement in brachytherapy.

**II. MATERIALS AND METHODS**

**A. Film calibration, scanning and processing**

A.1 Film dosimetry equipment and methodology

All film measurements were performed with Gafchromic EBT3 (Ashland ISP Advanced Materials, NJ, USA) from a single batch (Lot A05151203). Film scanning was performed in red-green-blue (rgb) format using a 48-bit (16-bit per channel) scanner (Epson Perfection V750 Pro) at 72 dpi, in transmission mode, with no colour or sharpness corrections, consistent orientation on the scanner and 48 hours from exposure to scanning, unless otherwise stated in the methodology. Dose-response calibration of the film was undertaken within FilmQAPro® software ([www.filmqapro.com](http://www.filmqapro.com), Ashland ISP Advanced Materials, NJ, USA, version 3.0.4835, released 28^th^ March 2013). A nominal 6 MV linear accelerator, traceably calibrated to a primary standard at the National Physical Laboratory (Teddington, UK) and measured using an ionisation chamber calibrated for absorbed dose to water, was used for film calibrations, and all test film dose exposures. Film strips of 10 x 5 cm were positioned on the central axis in a 10 x 10 cm field at 5cm depth in a 30 x 30 x 30 cm block of Solid Water (RMI457, Gammex, Wisconsin, USA) and each exposed to a different dose level: 0, 1, 2, 4, 8, 10, 15, 20 Gy. The average film pixel values in a 4 x 4 cm region centred on the axis of the beam were used to derive the average film response at each dose level.

Scanned images of irradiated films in TIFF format were converted to dose maps using both a single channel method (red channel) and a triple-channel method (red, green and blue channels), via FilmQAPro® software. The single-channel dose conversion utilises a simple calibration function; the red channel was chosen as this is the most commonly used in simple radiochromic film dosimetry since this wavelength has the highest absorption spectra.^(2)^ The multi-channel analysis method uses an algorithm described by Micke et al.^(8)^ to separate the scanned signal into a dose-dependent part and a dose-independent part. The algorithm essentially determines and subtracts a disturbance function that is independent of dose by fitting the measured colour signal to allowed colours in the dose-to-rgb signal calibration. Calibration functions of the form in equation 1 were derived for each colour channel.

X(D) = (a+bD)/(c+D), (1)

where X(D) is the scanner response at dose D, and a, b and c are the fitted function constants.

Reference dose films were also used for linear re-scaling of the calibration function to account for any system variations, such as scanner response, between test film and calibration film.

A.2 Investigation of post-irradiation film darkening

All film dosimetry requires careful consideration of post-irradiation film darkening. In this investigation, the net optical density of film test strips was determined as the ratio of the measured average pixel value in a 4 x 4 cm region of interest from the test film to the average pixel value from a fixed reference density sample (a film irradiated over 12 months ago, full expression of post-irradiation darkening having occurred). The test and reference density samples were scanned simultaneously, to account for any scanner-dependent variations with time. Nine Gafchromic EBT3 test films were irradiated to dose levels between 0 and 14 Gy, at 5 cm depth in a Solid Water phantom, and their optical density measured over a range 0.7 to 2277 hours (3 months) post irradiation. Individual film samples were scanned repeatedly for the various post-irradiation times. Additional control film samples exposed to the same dose level but not repeatedly scanned were compared after 3 months to ensure the test films were not affected by the repeated scanning.

**B. Evaluation of triple-channel dosimetry in test situations**

B.1 Lateral position of film on scanner

Four 4 x 4 cm EBT3 film pieces were exposed to 1, 8, 12 and 14 Gy respectively, at 5 cm depth in a Solid Water phantom. The films were cut in half to provide two identical film samples at each dose level. One of each dose level film was positioned along the central axis of the scanner, while the other piece was displaced laterally by 1, 2, 3, 4, 6, 7, and 9 cm, in both positive and negative directions. The lateral direction is defined as being perpendicular to the direction of travel of the scanning lamp, with zero lateral displacement being the centre of the scan plane. Scans were acquired at several lateral displacements. Film dosimetry using single-channel and triple-channel analysis was performed for each film piece in each scan. To account for any scan-dependent variations, the calculated film dose of the laterally displaced piece was corrected for any variations in the reported dose of the central piece. The resulting change in dose was a function of the lateral scanner effect only and this was compared for the two dosimetry methods.

B.2 Film surface perturbation

Three 4 x 4 cm Gafchromic EBT3 film samples were uniformly exposed to 0, 4 and 10 Gy respectively, at 5 cm depth in a Solid Water phantom. Each film had surface perturbations applied on one face: Scratches were made with medium hand-pressure using a solid block corner at one end and a thin layer of grease applied at the other end, typical of finger print marks. The change in colour signal caused by these disturbances was between 6 and 13% (at 16 bits per channel, in the red channel, for the 0 Gy film a maximum reduction from 40420 to 35280, and for the 10 Gy film, a maximum reduction from 13900 to 11860). Variations in surface perturbations between the films is inconsequential as this test examined the qualitative performance of single-channel and triple-channel dosimetry at each dose level. The films were scanned using the methodology described in A.1 and film-dose maps created using single-channel and triple-channel dosimetry. Dose profiles were taken through the two dose-maps and the effect of surface perturbations was compared.

B.3 Film active layer thickness

The measurement of the effect of variations in film active layer thickness on the scanned image and calculated film-dose is limited by difficulties in measuring the actual thickness of the active layer, sandwiched between two polyester layers. However, to test the concept of thickness compensation by multi-channel film dosimetry, the active layer of irradiated film samples were doubled through a process of de-lamination of irradiated EBT3 film and re-stacking to produce an effective double active layer, as shown in Fig. 1 . While there are significant uncertainties in the physical process of de-laminating and re-laminating, the process is sufficient to test the relative performance of single-channel and triple-channel dosimetry to significant variations in active layer thickness, accepting the additional error sources introduced by this process. One can then infer relative performance of the two systems to more modest variations that may be encountered in commercially available film. Four film samples were exposed uniformly to 1, 8, 12 and 14 Gy, at 5 cm depth in a Solid Water phantom. Each sample was cut into three pieces, two of which were carefully de-laminated. In de-laminating, the active layer remained adhered to one of the polyester sheets, the other being essentially clear, see Fig. 1. The polyester layers with the active layer attached were stacked together to produce a sample with an active layer of effectively double the original thickness. A scan was made of the two configurations: original film and re-stacked de-laminated with a double active layer. The film-dose for each of these at each dose level was determined using single-channel and multi-channel dosimetry.

**C. Evaluation of triple-channel dosimetry in clinical brachytherapy dose distributions**

Film measurement of a typical cervix treatment dose distribution using multiple dwells from a Nucletron microSelectron Ir-192 HDR source, in the vicinity of a Nucletron Interstitial Ring CT-MR treatment applicator (Nucletron, Netherlands), were obtained using a rigid support frame phantom consisting of a Solid Water structure in a water tank, described by Palmer et al.^(19)^ Gafchromic EBT3 films, 105 mm along x 80 mm wide, were positioned in the plane of the intrauterine tube, lateral through Point A and anterior to the treatment applicator, and exposed using a typical clinical treatment with an Ir-192 HDR source. With a prescription dose of 7 Gy to Point A, the dose range across the films was 0.3 to 13 Gy, with the highest doses at the corner of the film closest to the applicator. Films were converted to dose maps with single-channel and triple-channel dosimetry and compared to the Nucletron Oncentra Brachy (Nucletron, Netherlands) treatment planning system (TPS) (version v4.1.0.132) calculated 3D dose grid (1.0 mm resolution), using FilmQAPro software (Ashland ISP Advanced Materials, NJ, USA). Isodose overlay and gamma analysis^(20)^ were used to compare the film-dose and TPS calculated-dose maps. The gamma analysis passing rate was calculated using no threshold dose, 3% local normalisation and 2 mm criteria.

**D. Evaluation of proposed film dosimetry methodology applied to brachytherapy**

Lewis et al.^(17)^ have proposed an efficient methodology for film dosimetry in external beam radiotherapy, in which one reference dose film and an unexposed film are scanned simultaneously with the test film, to account for scanner-related variations and time-since-exposure darkening compensation from the calibration condition. In brachytherapy, the dose range across films may be significantly greater than that in external beam radiotherapy and the dose level for the reference dose film must be carefully selected. Ten Gafchromic EBT3 test films were each exposed to accurately known dose levels of 5, 7.5, 10 and 13 Gy, representative of dose ranges expected in brachytherapy film dosimetry applications, using a nominal 6 MV linear accelerator, at 5 cm depth in a Solid Water phantom. The film dose in 4 x 4 cm regions of interest at each dose level for each film was calculated using FilmQAPro software, triple-channel dosimetry, using 0 and 7.5 Gy and then 0 and 13 Gy reference doses for linear rescaling of the film calibration function. The average percentage difference of the film-calculated dose, at each dose level, from the anticipated dose, for the two calibration systems, was recorded and compared.

It has been anecdotally proposed that film scanning should be delayed for a time period of at least four times longer than the interval between the exposure of the test film and the calibration reference dose film, for typical external beam radiotherapy dosimetry, to ensure that time-after-exposure differences make insignificant dose error. Using data acquired from A.2 above, we test this proposition at dose levels up to 14 Gy, for brachytherapy film dosimetry applications, and at time intervals between test and reference films of up to 24 hours, much larger than values typically experienced in external beam dosimetry. There may be significant delays between the irradiation of test films on a brachytherapy unit and corresponding reference films from an external beam linear accelerator (used to provide uniform film-dose irradiation) due to practical equipment access issues within a radiotherapy department. In external beam therapy dosimetry, the test and reference films can be irradiated consecutively from the same radiation source.

In brachytherapy, due to the large dose range across the film, we propose that two reference calibration films, in addition to the unexposed film, are required to provide assurance of accuracy across the range of doses. We propose that one film be accurately exposed to the brachytherapy prescription dose (as this is generally of primary interest) and another to the expected maximum dose to be recorded on the film (to confirm accuracy across the dose range), in a nominal 6 MV linac, as described in Section II.A.1. The clinical test films described in Section II.C were exposed in this manner (7 Gy prescription dose and 13 Gy expected maximum to be recorded on the film). Fig. 2 depicts the arrangement of the test film, reference films, and unexposed film on the scanner. The unexposed film and the prescription dose film were used to re-scale the film calibration function within FilmQAPro, the latter being the dose level of greatest clinical significance. The high dose test film was used to validate the extrapolated calibration function for all dose levels across the film.

Errors in film dosimetry may be introduced if the film is not flat on the scanner. Curling of the film is more common with small film pieces, which are typically used in brachytherapy film dosimetry. Scans of a brachytherapy dose distribution imaged with a clear glass plate on top of the film to ensure perfect flatness and then with a significant induced curve (4 mm height from the glass plate at the centre of an 80 mm width film) were compared using isodose overlay.

Finally, scans of typical brachytherapy dose distributions were performed with and without an anti-reflective mat surrounding the films, which is often anecdotally proposed to minimise reflections from regions of the scanner plate not containing film. The film dose-maps with and without mats were compared.

**III. RESULTS**

**A. Film calibration**

Fig. 3 shows the pixel values in three colour channels as a function of irradiated dose for the Gafchromic EBT3 calibration film exposures, scanned 48 hours post exposure. The calibration function constants for equation 1, given in section II.A.1, were a=1.988, b=0.058, c=3.142 for the red channel, a=4.018, b=-0.005, c=6.791 for the green channel, and a=4.483, b=0.047, c=14.070 for the blue channel.

**B. Post-irradiation film darkening**

Fig. 4 shows the net optical density of EBT3 film as a function of time post irradiation over a range 0.7 to 2277 hours (approximately 3 months), at nine dose levels between 0 and 14 Gy. All curves fit a logarithmic function, of increasing gradient with dose level. Further analysis of this data is included in section III.G, below, as applied to a proposed methodology for film dosimetry in brachytherapy applications.

**C. Lateral displacement of film on scanner**

Fig. 5 shows film-dose calculated using triple-channel and single-channel dosimetry as a function of the lateral position of the film on the scanner, at dose levels 1, 8, 12 and 14 Gy. For both triple-channel and single-channel dosimetry, there is a lateral-scanner effect which increases the reported film dose with increasing lateral position of the film on the scanner. The effect is significantly larger for single-channel compared to triple-channel. The percentage increase in film-dose at 4 cm lateral distance compared to the film-dose on the central axis of the scanner, at 1 Gy, was 6% with single-channel dosimetry and 1% with triple-channel dosimetry, at 8 to 12 Gy was 7% with single-channel and 1% with triple-channel dosimetry, and at 14 Gy was 11% with single-channel and 2% with triple-channel dosimetry. At 9 cm lateral distance, at 1 Gy, the dose increase was 20% with single-channel dosimetry and 5% with triple-channel dosimetry, and at 14 Gy, the dose increase was 24% with single-channel dosimetry and 9% with triple-channel dosimetry. The results for positive and negative displacements in the lateral direction were consistent within experimental error.

**D. Film surface perturbation**

Fig. 6 shows film-dose profiles through three Gafchromic EBT3 film samples exposed to 0, 4 and 10 Gy, respectively, each with surface perturbations caused by the application of grease and scratches. Film dose calculated using triple-channel and single-channel dosimetry is compared in the figure. The surface perturbations have significantly greater effect on the reported dose using single-channel compared to triple-channel dosimetry, with the latter accurately reporting the expected dose values for all three films. Single-channel analysis reported significantly higher dose values due to the presence of grease and scratches.

**E. Film active layer thickness**

Table 1 gives the calculated dose values for the original film and ‘doubled active layer’ film scanned and analysed using single-channel and multi-channel dosimetry methods, at dose levels of 1, 8, 12 and 14 Gy. The process of de-laminating and re-stacking increases the noise in the scanned image, shown as an increase in the standard deviation of the sample region. However, it is clear that an effective doubling of the active layer is reported as an approximate doubling of the dose with single-channel dosimetry, but has much less effect on the dose reported by triple-channel dosimetry, with doses being approximately consistent with the original single active layer film. Triple–channel dosimetry is clearly less sensitive to active layer thickness variations than single-channel dosimetry.

**F. Measurement of clinical brachytherapy dose distribution**

Fig. 7 and 8 show isodose comparisons between treatment planning system (TPS) calculated dose planes and measured film isodoses using single-channel and triple-channel dosimetry. The corresponding gamma evaluation passing rates are provided in Table 2. Both single and triple channel dosimetry provides good agreement close to the applicator axis, up to 20 mm lateral (abscissa), corresponding to 3 to 13 Gy, and good agreement up to 105 mm from the applicator base (ordinate), corresponding to 5 to 13 Gy. While triple channel film dosimety maintains good agreement with TPS calculation across the entire film, the single channel analysis exhibits an increasing difference between film-dose and TPS-dose with increasing lateral distance (abscissa): This corresponds to increased lateral distance on the scanner. The gamma passing rates presented in Table 2 demonstrate dramatically improved agreement between film-dose and TPS-dose when triple-channel dosimetric analysis is used compared to single-channel. These results are consistent with the lateral scanner artefact presented in section III.C.

**G. Evaluation of proposed methodology for film dosimetry in brachytherapy**

Table 3 shows the average calculated film-dose at several known dose levels across a wide dose range from a series of ten test films. The primary calibration function was derived over a dose range 0 to 20 Gy. Within FilmQAPro® software, the calibration function was re-scaled to an unexposed film and a known dose level film, scanned within the same image as the test film. This allows correction for any scanner-related response changes since the film calibration. The calculated film-dose is forced into agreement with the expected dose at the reference dose level, with the percentage difference increasing at other dose levels. While maintaining the 0 to 20 Gy calibration function, two different sets of reference dose films, scanned with the test film, were used. With reference doses of 0 and 7.5 Gy a maximum discrepancy of 1.4% across the range 5 to 10 Gy, increasing to 2.3% at 13 Gy was found. Using reference doses of 0 and 13 Gy gave agreement of 0.1% at 13 Gy, but a maximum discrepancy of 2.1% across 5 to 10 Gy.

The results in section III.A have been used to determine the required delay before scanning of simultaneous test and reference films, such that post-exposure darkening of both films does not introduce significant uncertainty into the dosimetry results. The rate of change of optical density post exposure increases with dose level, hence the longest delay before scanning is required for the highest dose level. Considering the measured rate of change at 14 Gy exposure, Fig. 4, the required delay for typical brachytherapy dosimetry situations has been evaluated. A worst case delay between a brachytherapy test exposure and a reference film exposure from a different (external beam) treatment unit may be typically 24 hours. If the test film irradiation is performed at time t = 0 hours, and the reference film irradiation at t = 24 hours, for the same 14 Gy dose level, the difference in net optical density of the films due to post-exposure darkening would be 5% if scanned at 0.1 hour following the reference film exposure. This reduces to 3% at 1 hour, 1% at 12 hours, 0.6% at 48 hours, 0.4% at 72 hours, and 0.2% at 96 hours following reference film exposure. For a time difference of 6 hours between test and reference films, the net optical density difference between the films at a dose of 14 Gy is 3.8% at 0.1 hour post exposure, reducing to 0.3% at 24 hours. For a time difference of 2 hours, the difference in net optical density at 14 Gy is 2.8% at 0.1 hour, reducing to 0.3% at 8 hours post exposure.

Fig. 9 shows an isodose overlay comparison between film-dose maps from a brachytherapy dose distribution scanned with the film perfectly flat using a compression glass plate and with an induced significant curvature of the film (isodoses aligned at the right side of the image). There is a significant shift in the isodoses of the non-flat film. With the film curled and raised up from the scanner plate, there is an apparent increase in the pixel value and reduction in film-dose.

No significant difference was measured between films scanned with or without a surrounding anti-reflective mat on the scanner. A comparison between film-measured dose distributions of typical brachytherapy treatments, as discussed in Section III.C, scanned with and without the mat, compared using gamma analysis at 1% (local normalisation) and 1 mm had a passing rate of 99.2%.

**IV. DISCUSSION**

The post-irradiation darkening behaviour of Gafchromic EBT3 film has been shown to be a logarithmic function, continuing at least 3 months post-exposure, with the effect more significant at higher dose levels, shown in Fig. 4. These results differ from Borca et al.^(14)^ who considered post-irradiation coloration over 72 hours, and concluded net optical density stabilised after nearly 30 minutes at dose levels up to 2 Gy, and 2 hours would be sufficient to guarantee stability to perform analysis at studied doses up to 4 Gy. Theoretically, the film active layer polymer formed by exposure will continue to grow but the rate of growth will rapidly decrease as the distance from the growing polymer chain to the next available monomer increase. Growth will only occur on random occasions when enough thermal energy is acquired to bridge the gap. The results presented here demonstrate post irradiation colorisation is a continual, logarithmic function, as predicted by theory. It is important to appreciate that continual darkening of the film occurs, and films irradiated for purposes of reference dose calibration, scanned simultaneously with test films, must be irradiated at the same time, or the time difference recorded and sufficient time allowed between test and reference irradiations prior to scanning. The anecdotal recommendation of delaying scanning by a time four times the interval between test and reference films has been shown to be valid in this work up to 14 Gy and 24 hours between test and reference films, in order to reduce dose errors to less than 0.3%.

The lateral scanner artefact described by Menegotti et al.^(16)^ for doses up to 7 Gy, has been confirmed in this work and documentation of its effect extended up to 14 Gy, appropriate for brachytherapy film dosimetry. Menegotti et al.^(16)^ showed a change in pixel value in the range 9 to 19% depending on scanner model, for a 7 Gy exposure at 10 cm lateral to the central axis of the scanner. The results presented in the current work indicate a reported dose increase of 23% at 14 Gy, 9 cm lateral position, for single-channel film dosimetry. However, this effect is reduced to 9% with triple-channel dosimetry. The near linear array of monomer rods in the film means the amount of polarisation of light is different depending on whether the film is 0 or 90 degrees rotated on the scanner, and this may explain the difference in results between our work and Menegotti et al.^(16)^.

The advantage of triple-channel dosimetry over single-channel dosimetry in mitigating the effect of lateral scanner artefact has been demonstrated at dose levels and distributions typical for brachytherapy dosimetry. However, the useable scanner width is limited even with triple-channel dosimetry to less than ±4 cm width to reduce lateral scanner effect to less than 1%, with the Epson V750 Pro scanner (the useable width may increase with physically larger scanners). The effect of film surface perturbations and of variations in active layer thickness have been shown to be significantly reduced with triple-channel compared to single-channel dosimetry.

A proposed methodology for brachytherapy film dosimetry has been discussed, using three reference strips (including zero dose), rather than two (including zero dose) in order to confirm accurate dosimetry over the large dose ranges encountered in brachytherapy. We have also demonstrated that choice of reference film dose level for linear calibration re-scaling can improve the dose uncertainty at dose levels of interest. This is particularly important in brachytherapy dosimetry in which there is often very large dose ranges across films. We recommend to use a reference dose level for rescaling of the film response function around the dose level of particular interest, such as the prescription dose, rather than the maximum dose in the film, as recommended by Lewis et al.^(17)^ for external beam radiotherapy dosimetry applications, in which there is often a smaller dose range. We also recommend that film is perfectly flat on the scanner to provide reliable dosimetric results, avoiding changes in scanner response which may be due to variations in illumination, optical disturbances, and effects such as that described by Callier^(21)^ in which light changes from a collimated to a diffuse source. A compression glass plate positioned on top of the film is suggested to ensure sufficient flatness.

The proposed methodology for film dosimetry utilising the triple-channel algorithm implemented within FilmQAPro® software, as discussed in this work, has been applied to the measurement of dose distributions around clinical brachytherapy treatment applicators. Using gamma analysis to compare film-measured dose distributions with treatment planning system calculations, at criteria 3% local normalisation and 2 mm distance to agreement, over a region of interest of 105 x 80 mm (equivalent to 0.3 to 13 Gy dose range), gamma passing rates exceeding 90% for triple-channel dosimetry have been reported, but the passing rate reduced to exceeding 37% for single-channel dosimetry. (Due to very steep dose gradients, the position sensitivity of the gamma map is high, and in each case the relative position of the film-dose and plan-dose was optimised for maximum gamma pass rate). The significant reduction in performance of film dosimetry for brachytherapy with single-channel dosimetry compared to triple-channel dosimetry is likely to be primarily the result of lateral scanner artefact, which is mitigated with triple-channel dosimetry.

**IV. CONCLUSIONS**

The use of triple-channel film dosimetry in brachytherapy has been evaluated in both test cases and clinical dose distribution measurements. We have demonstrated that radiochromic film dosimetry with Gafchromic EBT3 film and a flatbed scanner is a viable method for brachytherapy dosimetry, and uncertainties may be reduced with triple-channel dosimetry and specific film processing methodologies. The separation of the scanner signal into dose and dose-independent parts via triple-channel dosimetry enables the mitigation of signal disturbances such as variations in film active layer thickness, film surface perturbations, and lateral scanner effect. The lateral effect is particularly significant for accurate dosimetry and must be considered in brachytherapy exposures to high dose levels, and even with triple-channel dosimetry limits the usable lateral region of the scan plane. The use of simultaneous scanning of calibration reference films with test films is advantageous to scale the pixel value for any scanner fluctuations, provided post-exposure darkening kinetics are accounted for between the two films. Darkening of the film continues after irradiation as a logarithmic function with time, at least up to 3 months. We have also demonstrated the importance of keeping film flat when scanning, an effect overlooked in previous recommendations on film dosimetry, but it is not necessary to use a non-reflective mat around the films.

**ACKNOWLEDGEMENTS**

The authors gratefully acknowledge Ashland ISP, USA, and Vertec, UK, for the supply of Gafchromic EBT3 film used in this investigation.

**REFERENCES**

1. Niroomand-Rad A, Blackwell C R, Coursey B M, et al. Radiochromic film dosimetry: recommendations of AAPM Radiation Therapy Committee Task Group 55. American Association of Physicists in Medicine Report no. 63. Med Phys. 1998;25:2093-115.

2. Devic S. Radiochromic film dosimetry: Past, present, and future. Phys Med. 2011;27:122-34.

3. Butson MJ, Yu PKN, Cheung T, Metcalfe P. Radiochromic film for medical radiation dosimetry. Mater Sci Eng. 2003;41:61-120.

4. Brown TAD, Hogstrom KR, Alvarez KL, Matthews KL, Ham K, Dugas JP. Dose-response curve of EBT, EBT2, and EBT3 radiochromic films to synchrotron-produced monochromatic x-ray beams. Med Phys. 2012;39:7412-7.

5. Alnawaf H, Peter KN, Butson M. Comparison of Epson scanner quality for radiochromic film evaluation. J Appl Clin Med Phys. 2012;13(5):314-21.

6. Matney JE, Parker BC, Neck DW, Henkelmann G, Rosen II. Evaluation of a commercial flatbed document scanner and radiographic film scanner for radiochromic EBT film dosimetry. J Appl Clin Med Phys. 2010;11(2):198-208.

7. Perez-Calatayud J, F. Ballester, R.K. Das, et al. Dose calculation for photon-emitting brachytherapy sources with average energy higher than 50 keV: Report of the AAPM and ESTRO. Med Phys. 2012;39:2904-29.

8. Micke A, Lewis DF, Yu X. Multichannel film dosimetry with nonuniformity correction. Med Phys. 2011;38:2523-34.

9. Mayer RR, Ma F, Chen Y, et al. Enhanced dosimetry procedures and assessment for EBT2 radiochromic film. Med Phys. 2012;39(4):2147-55.

10. Van Hoof SJ, Granton PV, Landry G, Podesta M, Verhaegen F. Evaluation of a novel triple-channel radiochromic film analysis procedure using EBT2. Phys Med Biol. 2012;57:4353-68.

11. Sim GS, Wong JHD, Ng KH. The use of radiochromic EBT2 film for the quality assurance and dosimetric verification of 3D conformal radiotherapy using Microtek ScanMaker 9800XL flatbed scanner. J Appl Clin Med Phys. 2013;14(4):85-95.

12. Palmer A, Bradley D, Nisbet A. Physics-aspects of dose accuracy in high dose rate (HDR) brachytherapy: source dosimetry, treatment planning, equipment performance and in vivo verification techniques. J Contemp Brachyther. 2012;4(2):81-91.

13. Devic S, Aldelaijan S, Mohammed H, Tomic N, Liang L-H, DeBlois F. Absorption spectra time evolution of EBT-2 model GAFCHROMIC^TM^ film. Med Phys 2010;37(5):2207-14.

14. Borca VC, Pasquino M, Russo G et al. Dosimetric characterization and use of GAFCHROMIC EBT3 film for IMRT dose verification. J Appl Clin Med Phys. 2013;14(2):158-71.

15. Martisikova M, Ackermann B, Jakel O. Analysis of uncertainties in Gafchromic® EBT film dosimetry of photon beams. Phys Med Biol. 2008;53:7013-27.

16. Menegotti L, Delana A, Martignano A. Radiochromic film dosimetry with flatbed scanners: A fast and accurate method for dose calibration and uniformity correction with single film exposure. Med Phys. 2008;35(7):3078-85.

17. Lewis D, Micke A, Xiang Y. An efficient protocol for radiochromic film dosimetry combining calibration and measurement in a single scan. Med Phys. 2012;39(10):6339-50.

18. Reinhardt S, Hillbrand M, Wilkens JJ, Assmann W. Comparison of Gafchromic EBT2 and EBT3 films for clinical photon and proton beams. Med Phys. 2012;39(8) 5257-62.

19. Palmer AL, Lee C, Ratcliffe AJ, Bradley D, Nisbet A. Design and implementation of a film dosimetry audit tool for comparison of planned and delivered dose distributions in high dose rate (HDR) brachytherapy. Phys Med Biol. 2013;58:6623-40.

20. Low DA, Harms WB, Mutic S, Purdy JA. A technique for the quantitative evaluation of dose distributions. Med Phys. 1998;25:656-61.

21. Chavel P, Lowenthal S. Noise and coherence in optical image processing. I. The Callier effect and its influence on image contrast. J Opt Soc Am. 1978;68:559-68.

**Figures and Figure legends**


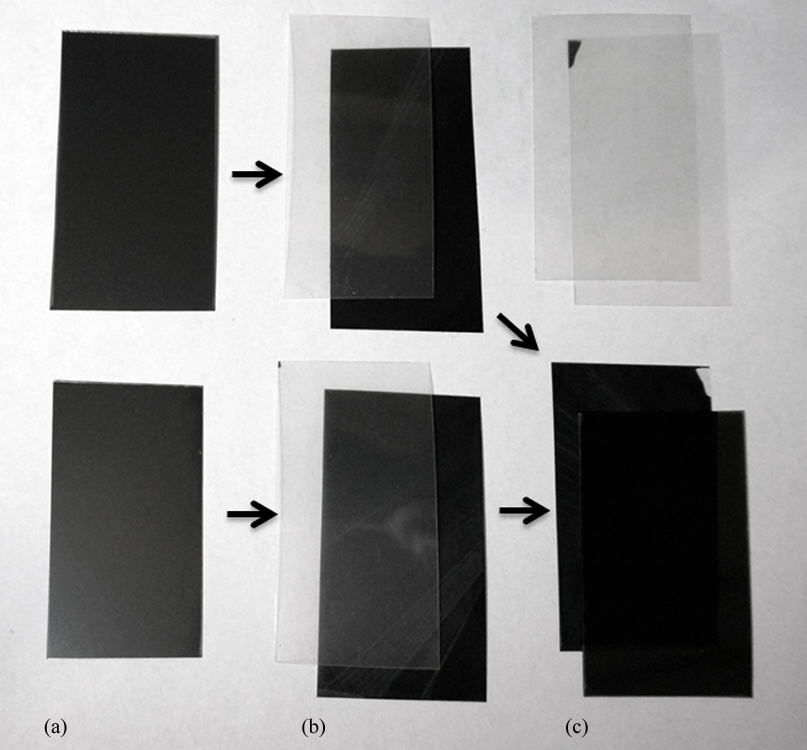


Fig.1. Process of delamination and re-stacking to produce a double-thickness active layer. (a) Original films, (b) de-laminated with active layer adhered to one polyester, (c) re-stacked with effective two active layer thickness.

**
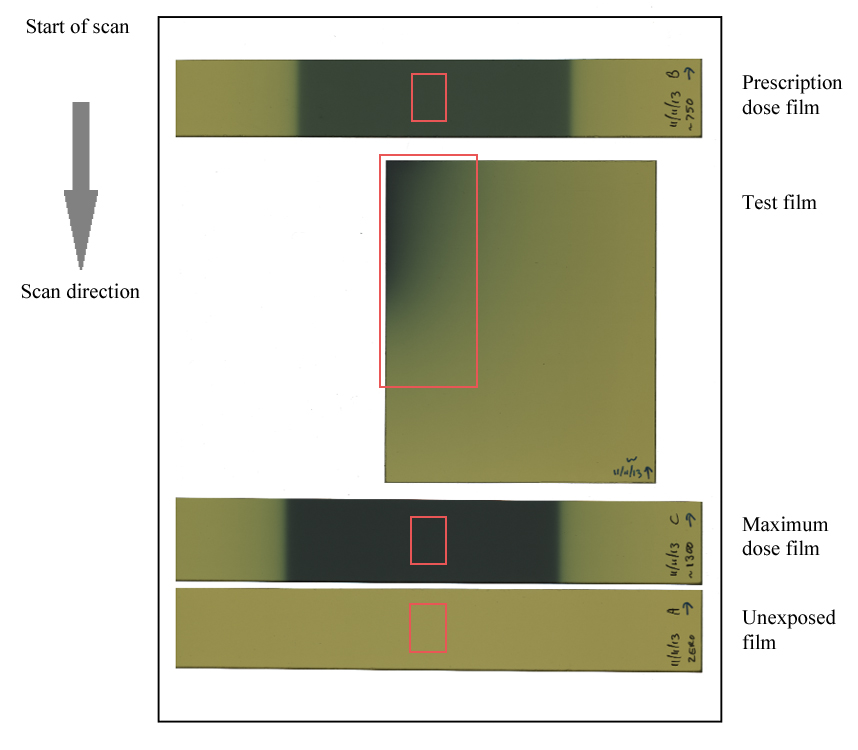
**

Fig. 2. Arrangement of films for simultaneous scanning. The regions of interest, shown as red rectangles in the figure, were placed centrally left-to-right in the scan window. For the clinical brachytherapy test film, the region of interest is in this case the corner of the film (which was closest to the clinical applicator and received the highest dose). This is aligned centrally with the regions used for calibration and validation.

**
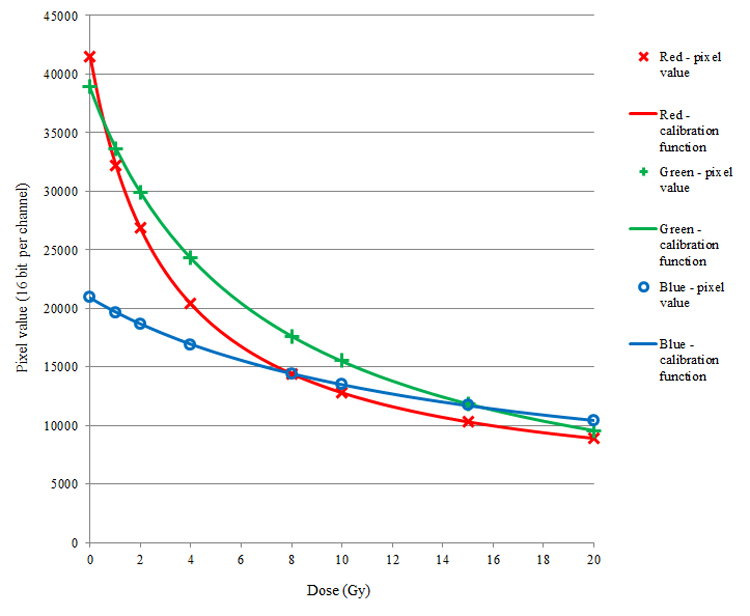
**

Fig. 3. Pixel value as a function of irradiated dose for Gafchromic EBT3 film scanned 48 hours post exposure, for red, green and blue channels, with fitted calibration functions (equation 1 in the text).


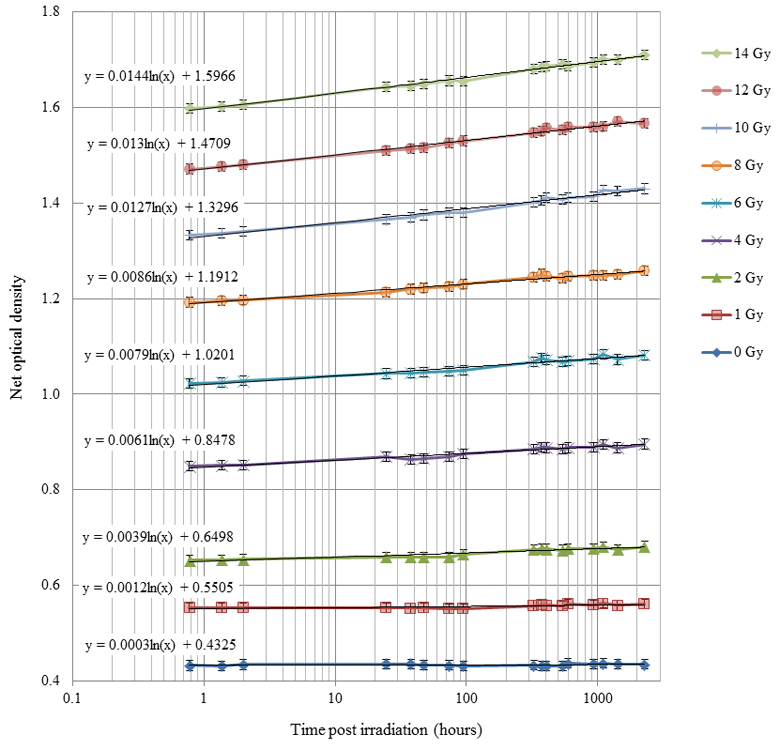


Fig.4. Gafchromic EBT3 film net optical density (as a ratio to a simultaneously scanned reference density sample) as a function of time post-irradiation, up to 3 months (2277 hours), over a dose range 0 to 14 Gy. (Error bars indicate one standard deviation of the sampled pixels).


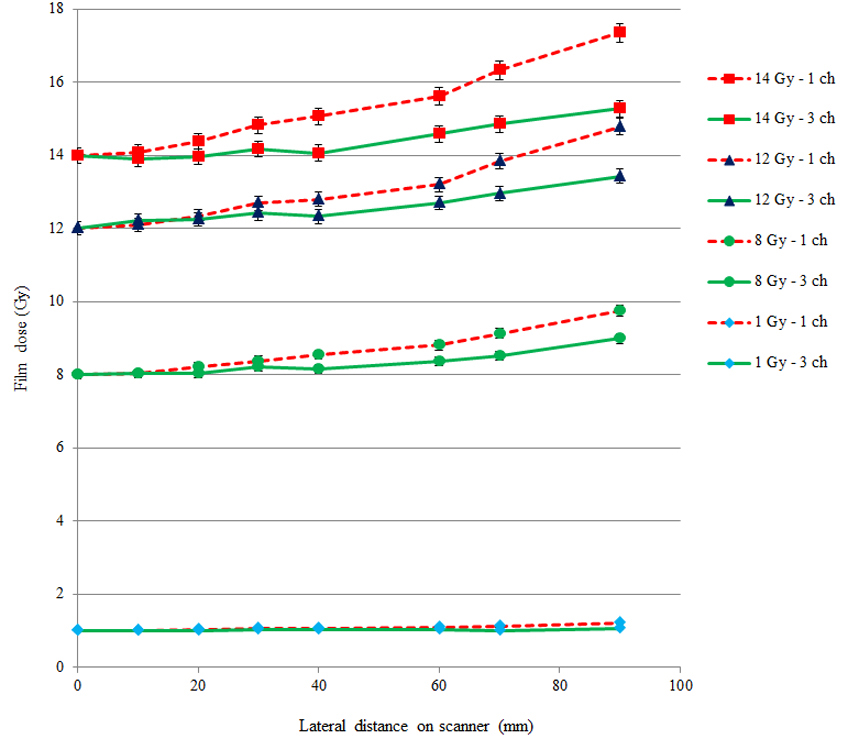


Fig.5. Change in film-dose as a function of lateral distance on scanner, using single-channel (dashed line) and triple-channel (solid line) film dosimetry, over range 1 to 14 Gy. (Error bars indicate one standard deviation of the sampled pixels).


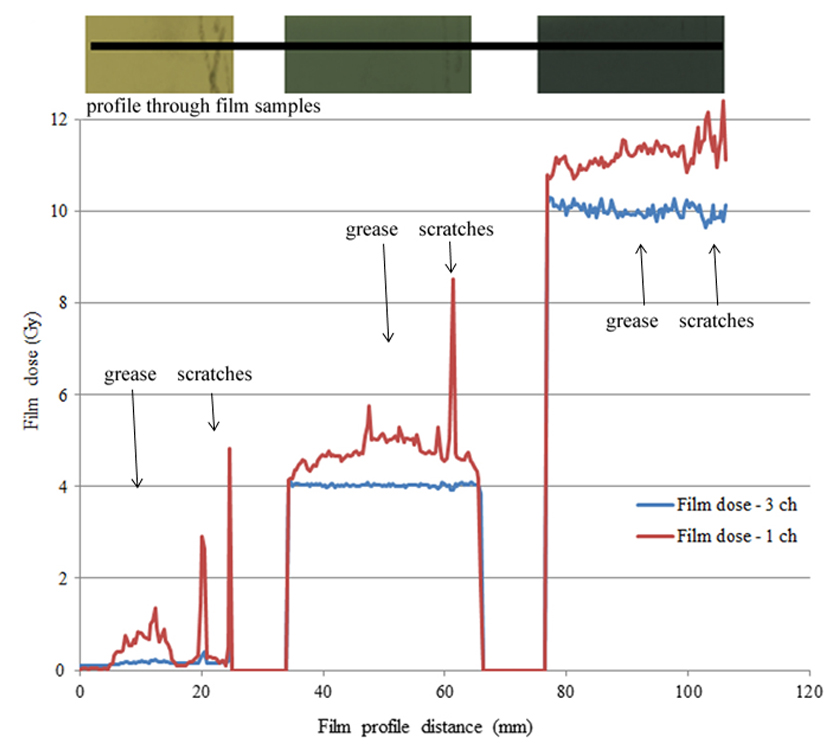


Fig. 6. Effect of film surface perturbation (grease and scratches) on calculated film-dose using single-channel and triple-channel dosimetry. Profile through three film samples irradiated to 0, 4 and 10 Gy each with grease and scratches.


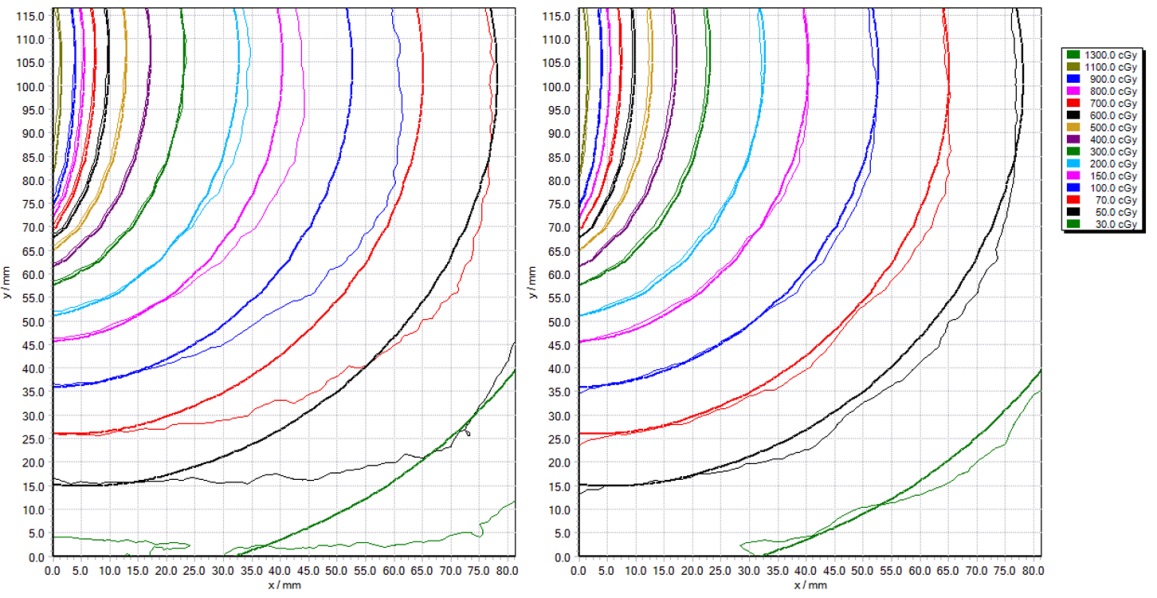


Fig. 7. Comparison of film-measured (thin lines) and treatment planning system calculated (thick lines) dose distributions from a typical clinical brachytherapy treatment, in a plane anterior to a cervix brachytherapy applicator, with (left) single-channel film dosimetry and (right) triple-channel film dosimetry. Abscissa aligned with lateral direction on the scanner, where x = 0 represents the middle of scanner.


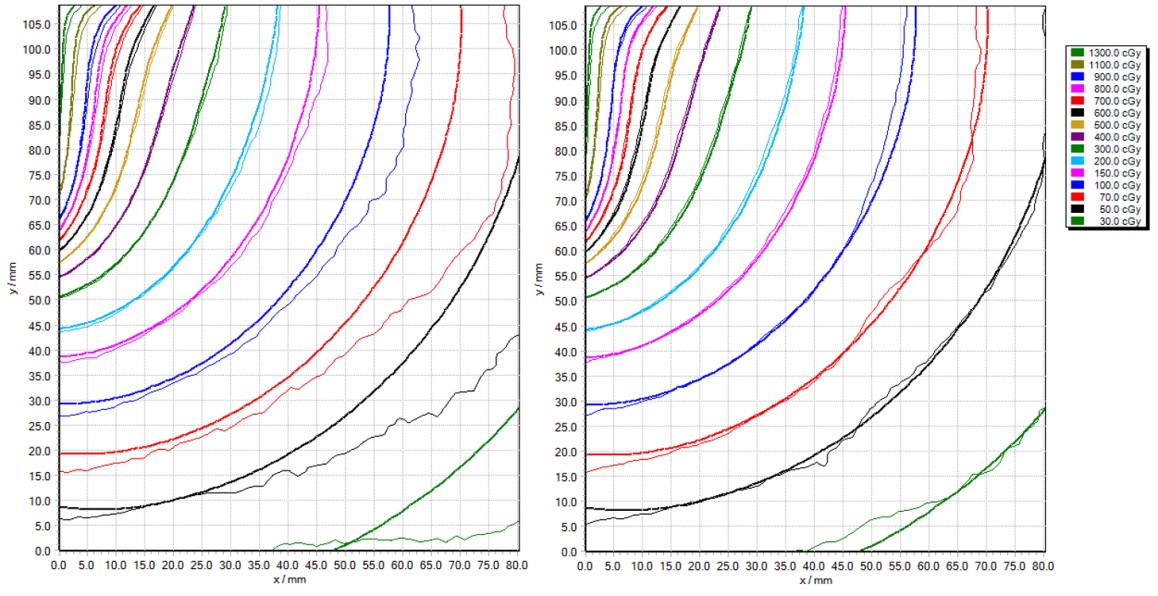


Fig.8. Comparison of film-measured (thin lines) and treatment planning system calculated (thick lines) dose distributions from a typical clinical brachytherapy treatment, in a plane lateral to a cervix brachytherapy applicator, with (left) single-channel film dosimetry and (right) triple-channel film dosimetry. Abscissa aligned with lateral direction on the scanner, where x = 0 represents the middle of scanner.


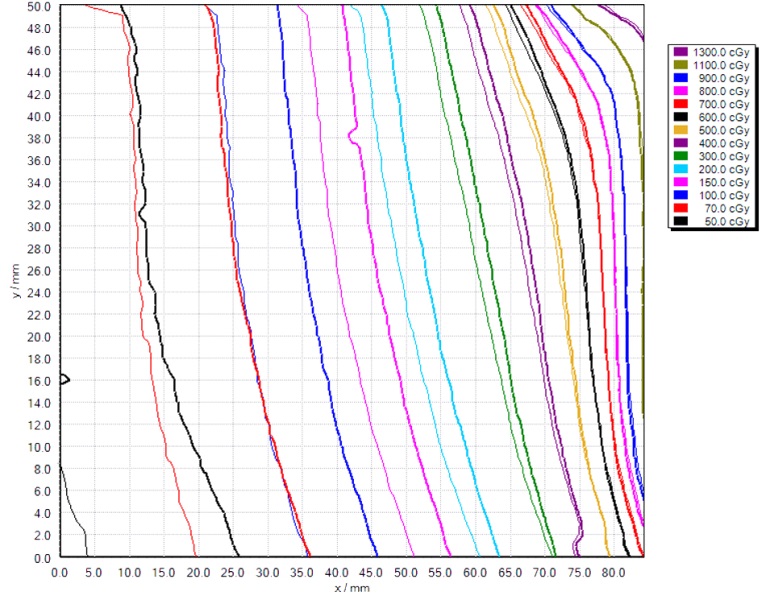


Fig.9. Isodose comparison of film dose from a typical brachytherapy dose distribution, with the film perfectly flat (thin lines) (compressed by glass plate) and with induced significant curvature (thick lines) (4 mm height of film from scan plane at the left of the figure, 0 mm height at the right). There is an apparent reduction in dose with the film raised from the scanner.

**Tables and captions**

Table. 1. Calculated film-dose for original and ‘double active layer’ films using single- and multi-channel analysis.

| Dose level (cGy) | Film dose from original film | | Film dose from de-laminated film and re-stacked with two active layers | |
| --- | --- | --- | --- | --- |
|  | Single-channel dosimetry (cGy) | Triple-channel dosimetry (cGy) | Single-channel dosimetry (cGy) | Triple-channel dosimetry (cGy) |
| 100 | 100.2 (±2) | 100.0 (±2) | 246.5 (±105) | 91.6 (±35) |
| 800 | 785 (±11) | 799 (±16) | 1681.3 (±269) | 866.2 (±118) |
| 1200 | 1166 (±26) | 1214 (±28) | 2452.1 (±204) | 1187.5 (±223) |
| 1400 | 1401 (±22) | 1400 (±35) | 2601.4 (±390) | 1426.5 (±261) |

Mean film-dose calculated using single- channel dosimetry and triple- channel dosimetry, for original Gafchromic EBT3 film and for delaminated and re-stacked EBT3 film producing an effective double thickness active layer, for doses in the range 1 to 14 Gy. One standard deviation of the pixel dose values in the regions of interest is shown in brackets.

Table. 2. Gamma analysis comparing film-measured with treatment planning system-calculated dose distributions, from a clinical cervix applicator.

| Region of interest (105 mm along x 80 mm away) | Gamma passing rate,  at 3% (local norm.) 2 mm, zero threshold | |
| --- | --- | --- |
|  | Single-channel film dosimetry | Triple-channel film dosimetry |
| Anterior to, and in the plane of, the intrauterine channel of cervix brachytherapy treatment applicator | 37.5% | 90.6% |
| Lateral to, and in the plane of, the intrauterine channel of a cervix brachytherapy treatment applicator | 50.1% | 95.2% |

Table. 3. Calculated average film-dose from ten test films using different calibration references.

| Film dose references for calibration function scaling | Percentage difference of calculated film-dose from actual irradiated dose, for each dose level | | | |
| --- | --- | --- | --- | --- |
|  | 5 Gy | 7.5 Gy | 10 Gy | 13 Gy |
| 0 and 7.5 Gy | +0.1% | +0.4% | +1.4% | +2.3% |
| 0 and 13 Gy | -1.0% | -2.1% | -1.1% | +0.1% |

The calculated average film-dose in a 4 x 4 cm region of interest using FilmQAPro® software, triple-channel dosimetry, using either 0 and 7.5 Gy or 0 and 13 Gy reference dose films, which are used for linear scaling of the film calibration functions to account for system variations compared to calibration film scans. The percentage difference of the film-dose to the actual irradiated dose is shown in the table.
